# Drug rash with eosinophilia and systemic symptoms to anti‐tuberculosis therapy: A retrospective review of inpatients at an academic medical centre in the United States

**DOI:** 10.1002/ski2.337

**Published:** 2024-02-28

**Authors:** Rodrigo A. Gutierrez, Maha Kazmi, Lindy Fox, Kanade Shinkai, Ryan Arakaki, Allison Dobry, Anna Haemel

**Affiliations:** ^1^ School of Medicine University of California San Francisco California USA; ^2^ Department of Dermatology University of California San Francisco California USA

## Abstract

This retrospective cohort study analyzes the presentation, diagnosis, and treatment outcomes of patients who developed drug rash with eosinophilia and systemic symptoms (DRESS) to tuberculosis (TB) therapy in a TB non‐endemic region. Anti‐TB agents represented 7.5% of all antimicrobial‐induced DRESS cases, and rifampin was the most commonly implicated agent among drugs used to treat TB.

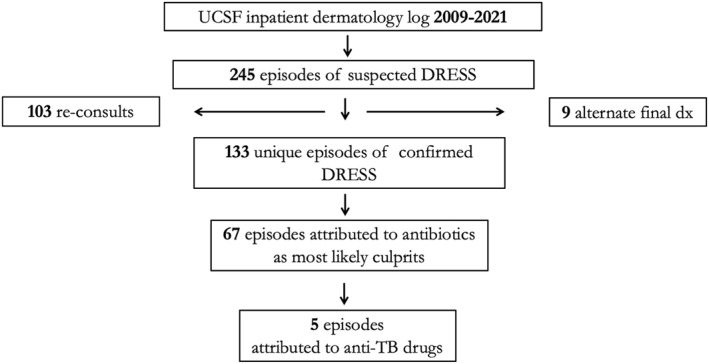

Dear Editor,

Drug rash with eosinophilia and systemic symptoms (DRESS), also known as drug‐induced hypersensitivity syndrome (DIHS) represents a severe, life‐threatening reaction that can occur in the setting of various medication exposures, including antimicrobials used to treat tuberculosis (TB).^1^, ^2^, ^3^ Because the implication of anti‐TB therapy in DRESS may result in pivotal changes to treatment plan, including withdrawal of suspected culprit agents and/or addition of immunosuppression, we sought to better characterize the clinical course of DRESS to anti‐TB medication at a U.S. academic hospital.

Cases consistent with DRESS were identified from the UCSF dermatology inpatient log from December 2009 to February 2021 in which the final diagnosis contained search terms DRESS, drug‐induced hypersensitivity reaction (DIHS), or ‘drug’ and ‘eosinophilia’ in the same entry, resulting in 245 cases (Supporting Information S1: Appendix). One hundred and three re‐consults for the same episode of DRESS were subsequently removed, along with 9 cases with an alternative final diagnosis, yielding 133 cases. Sixty‐seven of these episodes were attributed to antibiotics. Five of these cases consisted of anti‐TB medications as leading culprit agents, including rifampin, isoniazid, pyrazinamide, and ethambutol (RIPE). Culprit agents were determined by the dermatology consult team by drug exposure review and timing of reaction onset.

The cohort had a mean age of 59.4 (range 15–87) and was 4/5 (80%) male. All patients presented with a morbilliform rash characteristic of DRESS. Additionally, patients had hepatic involvement in 5/5 (100%), fevers in 4/5 (80%), renal involvement in 1/5 (20%), and pulmonary involvement in 1/5 (20%), and lymphadenopathy in 1/5 (20%). Four patients (80%) also had new peripheral eosinophilia, with the remaining patient experiencing possible blunting of eosinophilia secondary to existing steroid therapy for TB meningitis.

Of these five cases, rifampin and isoniazid were equally likely culprit agents in two cases, rifampin and ethambutol in one, rifampin alone in one, and isoniazid alone in the final case, with less likely putative drugs belonging to different classes (Table 1). All five cases were exposed to the four agents included in RIPE therapy, with rifampin being implicated as a first or equally likely culprit agent in 80% of cases, isoniazid in 60%, and ethambutol in 20%.

**TABLE 1:** Analysis of culprit agents and clinical presentation by case and number of exposures.

Suspected culprit agents and clinical course							
Case	Most likely agent (#1)	Agent #2	Agent #3	Least likely agent (#4)	RegiSCAR^a^	Exposure length (days)	Admission length (days)
1	Rifampin & isoniazid		Pyrazinamide	Ethambutol	4	39	62
2	Rifampin				5	146	6
3	Isoniazid	Rifampin	Phenytoin		3	49	78
4	Rifampin & isoniazid				4	59	108
5	Rifampin & ethambutol		Isoniazid		6	8	45

Suspected culprit agents versus exposures							
Drug	Total exposures	1st or equally likely culprit	Ratio of suspected culprit agent to exposure				
Rifampin	5	4	80%				
Isoniazid	5	3	60%				
Ethambutol	5	1	20%				
Pyrazinamide	5	0	0%				

^a^ Registry of severe cutaneous adverse reaction scoring system.

In the treatment of these reactions, suspected culprit agents were stopped in all five cases, with four patients also receiving oral prednisone therapy with a duration of 3, 12, 20, and 56 weeks, respectively (mean 27.8 weeks). The presence of underlying TB infection impacted the steroid regimen in dose or duration in all five cases (100%), resulting in a taper courses that started at lower doses or were shorter than would otherwise be clinically indicated in four patients, and no systemic corticosteroid therapy at all for the remaining patient. Despite concern for clinical deterioration after starting prednisone, there were no reported infection exacerbations due to the steroid therapy. All five patients survived hospitalization and were stable upon discharge.

This cohort underscores the burden of DRESS caused by antimicrobial pharmacotherapy in hospitalized patients, with more than 50% of cases being attributable to these medications. Unlike other frequent inducers of hypersensitivity reactions such as anticonvulsants or xanthine oxidase inhibitors, antimicrobial‐induced DRESS represents a unique therapeutic challenge, as affected patients often have a concomitant infection that can be life threatening, particularly if antibiosis is withdrawn.^4^ TB further distinguishes itself among these infections by the number of agents required to treat it and the duration of therapy necessary to resolve the infection, thus exposing patients to multiple axes of potentially lethal disease processes. Anti‐TB agents were implicated in 7.5% of all antimicrobial‐induced cases of DRESS during our study period, highlighting the role of these regimens in inducing DRESS, even in a TB non‐endemic area. Notably, steroid therapy did not result in exacerbation of infection in this cohort, with all patients surviving hospitalization. Relative to the number of exposures, rifampin was the most likely anti‐TB medication to be implicated as a first or equally likely culprit agent, followed by isoniazid and ethambutol. Notably, pyrazinamide was not deemed a most likely culprit in any case despite being a component of the medication regimen in all five.

Larger studies are needed to further clarify typical systemic manifestations and imminent risk of exacerbating underlying infection with systemic steroid therapy. While limited by a small sample size at a single institution, this study may guide inpatient dermatologists during crucial moments in the management of DRESS in the setting of underlying TB infection.

## CONFLICT OF INTEREST STATEMENT

Dr. Haemel is a consultant to Guidepoint LLC and to CSL Behring.

## AUTHOR CONTRIBUTIONS

**Rodrigo A. Gutierrez**: Conceptualization (equal); data curation (equal); formal analysis (equal); investigation (equal); writing – original draft (equal); writing – review & editing (equal). **Maha Kazmi**: Writing – review & editing (equal). **Lindy Fox**: Data curation (equal). **Kanade Shinkai**: Data curation (equal). **Ryan Arakaki**: Data curation (equal). **Allison Dobry**: Data curation (equal). **Anna Haemel**: Conceptualization (equal); data curation (equal); formal analysis (equal); investigation (equal); methodology (equal); project administration (equal); supervision (equal); validation (equal); writing – review & editing (equal).

## FUNDING INFORMATION

This article received no specific grant from any funding agency in the public, commercial, or not‐for‐profit sectors.

## ETHICS STATEMENT

Reviewed and approved by UCSF IRB; approval #11‐05526.

## Supporting information

Supporting Information S1

## Data Availability

The data underlying this article will be shared on reasonable request to the corresponding author.
