# Supplementary material for: Drug rash with eosinophilia and systemic symptoms to anti‐tuberculosis therapy: A retrospective review of inpatients at an academic medical centre in the United States
Source: Skin Health Dis. 2024 Feb 28;4(2):e337. doi: 10.1002/ski2.337 (PMC10988693; doi:10.1002/ski2.337)
Supplement: Supplementary file 1 — Supporting Information S1 [file SKI2-4-e337-s001.docx]

**Appendix: Determination of study population**

Data obtained from UCSF inpatient electronic medical records.
